# Effectiveness of a Numerical Problem-Solving Module in Enhancing Renal Physiology Comprehension

**DOI:** 10.7759/cureus.110591

**Published:** 2026-06-10

**Authors:** Mayank Agarwal, Manish Goyal, Priyadarshini Mishra

**Affiliations:** 1 Physiology, All India Institute of Medical Sciences, Raebareli, Raebareli, IND; 2 Physiology, All India Institute of Medical Sciences, Bhubaneswar, Bhubaneswar, IND

**Keywords:** competency-based education, medical education, numerical analysis, renal physiology, teaching methods

## Abstract

Introduction

Renal physiology is integral to medical education but poses challenges due to its abstract and quantitative nature. Traditional didactic teaching methods often do not foster a deep understanding of key concepts. With the advent of competency-based medical education in India, there is a need for innovative approaches to enhance conceptual understanding and analytical reasoning. This study aims to evaluate the effectiveness of a numerical problem-solving module in enhancing first-year medical students’ understanding of renal physiology.

Methods

We conducted a quasi-experimental intervention study with pre- and post-test paired assessments among first-professional medical students in the Department of Physiology at the All India Institute of Medical Sciences, Bhubaneswar, India. The intervention consisted of a small-group discussion of a 20-question renal physiology numerical problem-solving module that followed traditional lectures. The numerical module covered key aspects of renal function, including volume of distribution, clearance, glomerular filtration rate and renal blood flow calculations, tubular processing, and acid-base balance. We administered pre- and post-tests consisting of 17 multiple-choice questions (MCQs) via Google Forms (Google LLC, Mountain View, CA, USA). We used MCQ scores to assess quantitative performance. Item analysis was performed for both pre- and post-test MCQs. We collected students’ perceptions using a validated questionnaire. Among 113 students, only 92 students attempted both pre- and post-tests, while 100 students anonymously submitted the complete questionnaire. We used a paired t-test to compare related groups. Statistical significance was set at p ≤ 0.05.

Results

The post-test score (12.5 ± 2.3; 73.8 ± 13.5%) showed a significant improvement (p < 0.001) compared with the pre-test scores (11.3 ± 2.4; 66.6 ± 14.4%). Item analysis revealed that pre-test low achievers demonstrated significantly higher post-test scores (8.1 ± 1.7 versus 11.8 ± 2.6, p < 0.001), whereas high achievers showed no significant change. Students’ feedback strongly supported the incorporation of numerical problem-solving modules into the curriculum, highlighting greater engagement, deeper understanding, and enhanced peer collaboration.

Conclusion

The numerical problem-solving module significantly enhanced students’ understanding of renal physiology, particularly benefiting low achievers. This study underscores the potential for numerical problem-solving to become a standard component of medical education, fostering analytical skills and confidence.

## Introduction

Renal physiology is a critical component of medical education, essential for both academic success and clinical competence [1]. First-year medical students must understand complex concepts in renal physiology, such as volume of distribution, glomerular filtration rate (GFR), renal blood flow (RBF), and acid-base balance [2,3]. These topics are essential for understanding renal disorders but are challenging due to their abstract and quantitative nature [4]. Traditional teaching methods, such as didactic lectures, may not adequately address these challenges, leaving students struggling to grasp concepts.

The competency-based medical education (CBME) framework introduced by the National Medical Commission of India emphasizes analytical reasoning and application-based learning [3]. Globally, medical education systems are increasingly integrating active learning and problem-solving modules into undergraduate curricula to better prepare students for clinical practice [5-8]. However, the undergraduate medical curriculum often lacks numerical problem-solving tailored to renal physiology.

Introducing a dedicated module for numerical problem-solving in renal physiology could address several critical needs in medical education. Such a module would enable students to apply theoretical concepts to realistic scenarios, deepening their understanding of complex physiological mechanisms. Furthermore, this approach could foster greater confidence in mastering challenging renal physiology concepts and align with CBME’s broader goals.

This shift toward CBME reflects a growing recognition that modern medical education must equip students not only with factual knowledge but also with the analytical tools to apply it in clinical settings. In this context, conceptual understanding means comprehension of the underlying principles, mechanisms, and relationships of a concept, while analytical reasoning means applying logic to interpret, analyze, and solve problems using available information. By integrating numerical problem-solving exercises, students may develop both conceptual understanding and analytical reasoning, thereby promoting deeper learning and improving their readiness for clinical decision-making.

Although active learning strategies have been shown to improve engagement and retention [5,6], research on integrating numerical problem-solving into renal physiology is scarce. Addressing this gap is crucial given the growing emphasis on quantitative reasoning in medical practice.

This study aimed to evaluate the effectiveness of a numerical problem-solving module in improving first-year medical students’ understanding of renal physiology. By addressing gaps in traditional teaching strategies and incorporating student feedback, this research offers valuable insights into curriculum development and the potential integration of numerically focused modules into medical education.

## Materials and methods

Study design

This was a quasi-experimental educational intervention study with pre- and post-test assessments.

Study setting

This quasi-experimental study was conducted in the Department of Physiology at All India Institute of Medical Sciences (AIIMS), Bhubaneswar, with data collected in January 2025. Approval from the Institutional Ethics Committee of AIIMS Bhubaneswar was obtained prior to the study.

Sample size

A convenience sampling method was used. The sample size was calculated later using the single proportion formula as follows: $n = \frac{N}{1 + N(\mathrm{MOE})^{2}}$, where N is the total number of first-year Bachelor of Medicine and Bachelor of Surgery (MBBS) students in our institute (125), MOE is the margin of error (0.1, maximum permissible for an exploratory study), and n is the desired sample size. Assuming that 50% of the subjects in the population have the factor of interest, a population size of 125, and an expected response rate of 100%, the study would require a sample size of 55 to estimate the expected proportion with 10% absolute precision and 95% confidence interval [9].

Inclusion and exclusion criteria

Inclusion criteria were first-year professional students enrolled in the MBBS course at AIIMS, Bhubaneswar, who voluntarily agreed to participate in this study.

Students with incomplete Google Forms (Google LLC, Mountain View, CA) submissions or who did not complete both pre- and post-test assessments were excluded.

Numerical-problem-solving module development

A module of 20 numerical-based questions covering most aspects of renal function was developed (Appendix A). The concepts covered included the volume of distribution, clearance calculation, determination of GFR and RBF, tubular processing of substances, and acid-base balance. The module contained questions that mimicked real-world data and case scenarios, were designed for educational purposes, and were intended to encourage peer collaboration. The authors verified content validity through independent reviews by subject-matter experts and ensured accuracy by solving all problems.

Pre-test and post-test multiple-choice questions (MCQs) preparation

The authors independently prepared MCQs for pre- and post-test assessments. From the initial pool, 20 MCQs were selected based on face validity and topic alignment.

A panel of 10 reviewers, including postgraduate trainees and postgraduate-qualified tutors from the Department of Physiology at AIIMS Bhubaneswar, reviewed these 20 MCQs to assess clarity, absence of ambiguity, and alignment with the study’s theme. MCQs with a content validity index of at least 0.8 were selected for assessment. Of the 20 MCQs, 17 met this criterion (Appendix B).

We ensured balanced difficulty by using the mean number of correct responses per MCQ across reviewers. Across all 10 reviewers, the mean number of correct responses per MCQ was comparable between the pre-test (5.7 ± 2.0) and the post-test (5.5 ± 2.2) (p = 0.672, paired t-test), indicating that the difficulty of the MCQs in both sets was equivalent.

The pre-test contained eight numerical MCQs and nine related theoretical MCQs. The post-test contained six numerical MCQs and 11 related theoretical MCQs. The difference in numerical MCQ numbers in the pre- and post-test arose due to selection for topic alignment and validity selection. All MCQs had four options, with only one correct answer.

Questionnaire preparation

To assess students’ perceptions of the renal numerical module, the authors developed and content-validated a 10-item closed-ended questionnaire rated on a 5-point Likert scale, supplemented by a single open-ended question to collect qualitative feedback (see Appendix C).

Study procedure

The study began after the renal physiology lectures concluded. A pre-test of 17 MCQs was administered to students during a tutorial session via Google Forms. The pre-test was immediately followed by a 90-minute small-group discussion on the numerical problem-solving module, with groups of 25 students. The discussion primarily focused on solving numerical problems. Each group was assigned a trained tutor to facilitate discussion and guide students through numerical problems in renal physiology. All tutors were post-MBBS trainees who had requisite experience to teach renal physiology to first-year MBBS students. Students were informed about the session but not about the test.

**Figure 1:**
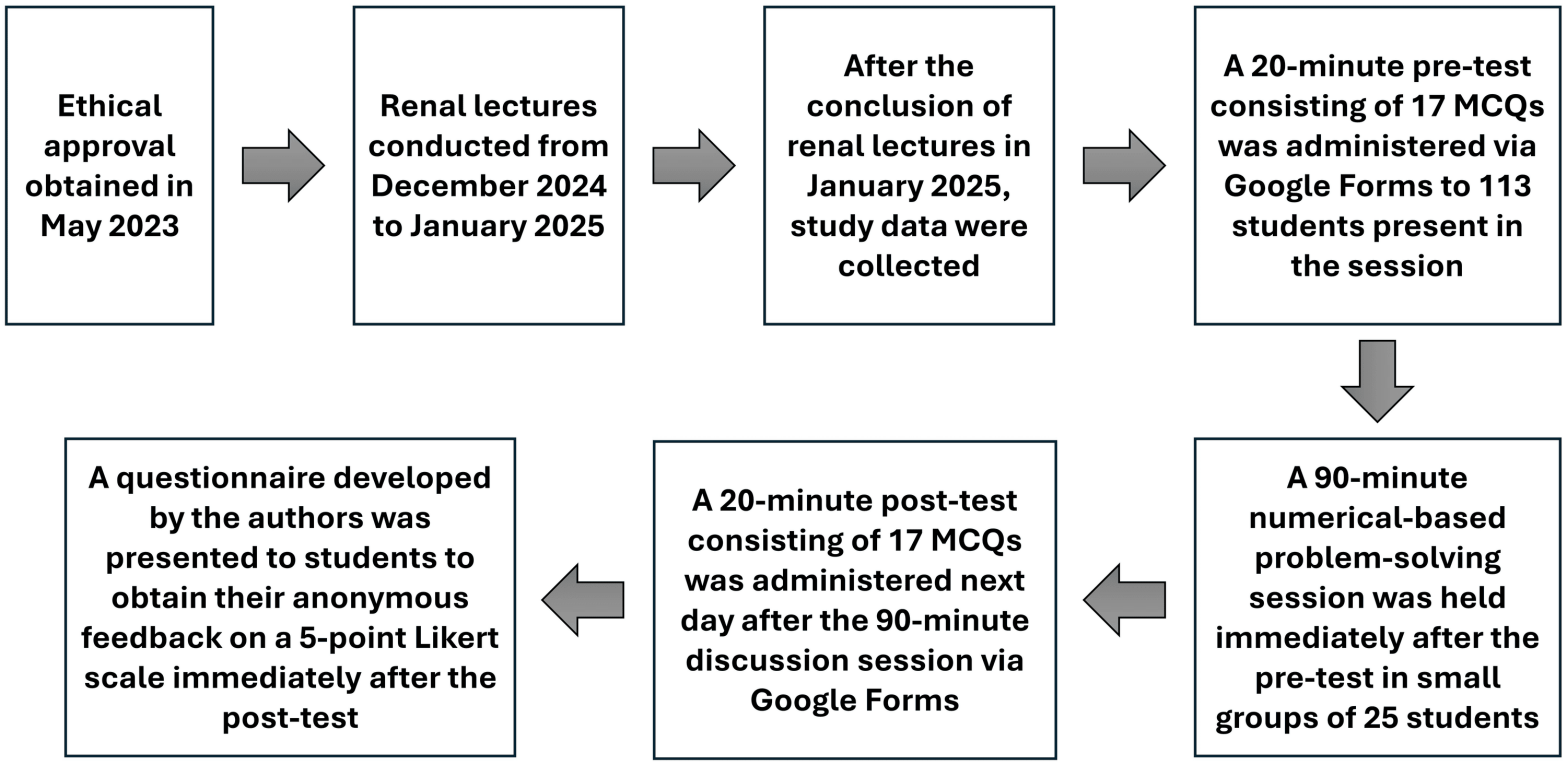
A visual representation of the study timeline MCQs: multiple-choice questions The figure was created by the authors using Microsoft PowerPoint (Microsoft Corp., Redmond, WA, USA).

The next day, a post-test of 17 MCQs, matched in difficulty and topic coverage to the pre-test, was administered via Google Forms. Student feedback was also collected anonymously through the same platform. Each test was timed for 20 minutes, with one mark awarded for each correct answer and no penalty for incorrect answers. A visual representation of the study timeline is presented in Figure 1.

**Figure 2:**
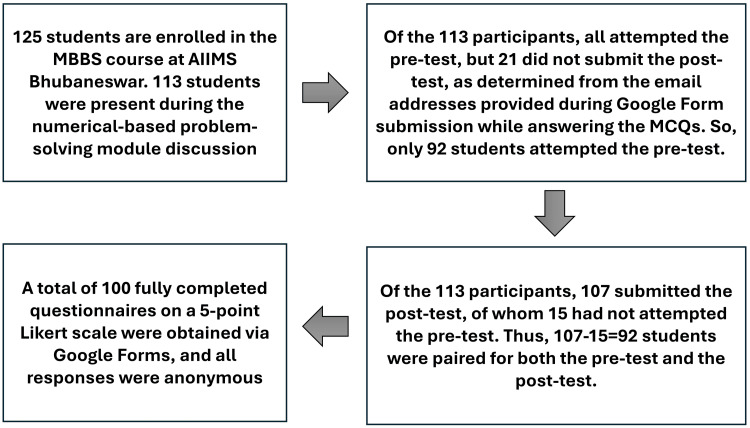
Flow diagram showing sample selection for item analysis, score comparison, and analysis of questionnaire responses MBBS: Bachelor of Medicine and Bachelor of Surgery; MCQs: multiple-choice questions The figure was created by the authors using Microsoft PowerPoint (Microsoft Corp., Redmond, WA, USA).

Among 125 first-year MBBS students enrolled at AIIMS Bhubaneswar, 113 were present and participated in the numerically based renal physiology discussion session. Of these, 92 students completed both the pre-test and the post-test. Additionally, 100 students submitted completely anonymous responses to the questionnaire administered through Google Forms. Figure 2 illustrates the process used for selecting the sample (n = 92) for item analysis and score comparison. It also depicts the selection of the sample (n = 100) included in the analysis of questionnaire responses obtained using a 5-point Likert scale.

Item analysis of MCQs

The pre- and post-test MCQs were analyzed for Difficulty Index (DifI), Discrimination Index (DI), and Distractor Effectiveness (DE) [10]. The top 27% (n = 25) students with the highest marks were classified as high achievers, while the bottom 27% (n = 25) with the lowest marks were classified as low achievers [10]. DifI was calculated as $\frac{(H + L)\times 100}{N}$. DI was calculated as $\frac{2(H - L)}{N}$, where H is the number of high achievers who answered the MCQ correctly, L is the number of low achievers who answered it correctly, and N is the total number of students in both groups [10]. A distractor in an MCQ was classified as a non-functional distractor (NFD) if it was selected by fewer than 5% of students [10]. DE was determined by the number of NFDs per MCQ: 100% for 0 NFDs, 66.7% for 1 NFD, 33.3% for 2 NFDs, and 0% for 3 NFDs [10].

Statistical analysis

Data were tabulated in Excel 365 (Microsoft Corp., Redmond, WA, USA) and analyzed in IBM SPSS Statistics for Windows, Version 27 (Released 2019; IBM Corp., Armonk, New York, United States). Results were summarized using the mean ± standard deviation (SD), median (interquartile range (IQR)), percentages, and frequencies. Normality was assessed with the Shapiro-Wilk test. A paired t-test was used to compare related groups. A two-tailed unpaired t-test or a Mann-Whitney U test was used to compare unrelated groups. Statistical significance was set at p ≤ 0.05, with a 95% confidence interval.

## Results

An analysis of students’ responses (n = 92) to MCQs showed a significant increase in the mean post-test score (12.5 ± 2.3) compared with the mean pre-test score (11.3 ± 2.4) (p < 0.001). Pre-test scores ranged from 4 to 16 out of 17, whereas post-test scores ranged from 6 to 17.

Table 1 presents the percentage scores for the pre- and post-tests. Overall, a significant increase in percentage scores was observed across all MCQs, including both numerical and theoretical items.

**Table 1:** Comparison of pre-test and post-test percentage scores MCQs: multiple-choice questions; paired t-test was used; n = 92

	Pre-test score in percentage	Post-test score in percentage	p
For all 17 MCQs	66.6 ± 14.4%	73.8 ± 13.5%	<0.001
For numerical MCQs only	70.7 ± 16.6%	75.9 ± 16.8%	0.045
For theoretical MCQs only	62.9 ± 16.6%	72.6 ± 14.4%	<0.001

The item analysis matrices showed no statistically significant differences between the pre-test and post-test. The mean DifI increased from 64.8 ± 21.2 in the pre-test to 71.7 ± 25.0 in the post-test (p = 0.396, unpaired t-test), while the mean DI changed marginally from 0.34 ± 0.19 to 0.31 ± 0.18 (p = 0.628, unpaired t-test). Similarly, the median DE decreased from 66.6 (33.3-83.4) to 33.3 (0.0-66.6), but the difference was not statistically significant (p = 0.092, Mann-Whitney U test).

The low-achieving students in the pre-test demonstrated significantly higher post-test scores, whereas the high-achieving group showed no significant difference (Table 2).

**Table 2:** Comparison of pre- and post-test scores of low and high achievers An unpaired t-test was used.

	Pre-test score (%)	Post-test score (%)	p
Pre-test low achievers group (n = 25)	8.1 ± 1.7 (47.8 ± 9.8%)	11.8 ± 2.6 (69.7 ± 15.3%)	<0.001
Pre-test high achievers group (n = 25)	13.9 ± 0.9 (81.9 ± 5.1%)	13.7 ± 1.6 (80.5 ± 9.1%)	0.508

The DE decreased significantly in the low-achievers group from pre-test to post-test, whereas it decreased non-significantly in the high-achievers group (Table 3).

**Table 3:** Comparison of pre- and post-test DE of low and high achievers DE: Distractor Effectiveness; Mann-Whitney U test was used.

	Pre-test DE	Post-test DE	p
Pre-test low achievers group (n = 25)	66.6 (50.0-100.0)	33.3 (33.3-66.6)	0.009
Pre-test high achievers group (n = 25)	33.3 (0.0-66.6)	0.0 (0.0-33.3)	0.394

Feedback from 100 students, in response to 10 closed-ended questions on a 5-point Likert scale, was collected via Google Forms. Because the feedback was completely anonymous, it cannot be determined whether these students attended both the pre- and post-test. The feedback questionnaire demonstrated good internal consistency, with a Cronbach’s alpha of 0.902. Table 4 summarizes the response frequencies and the median (IQR) scores for the 10 closed-ended questions.

**Table 4:** Frequency of responses and the median scores for the 10 closed-ended questions GFR: glomerular filtration rate; RBF: renal blood flow; n = 100

Questions	Strongly agree (5 points)	Agree (4 points)	Neutral (3 points)	Disagree (2 points)	Strongly disagree (1 point)	Median (IQR)
Did solving numerical problems reinforce your overall understanding of renal physiology?	65	23	10	2	0	5 (4-5)
Did the numerical problem-solving exercise enable deeper exploration of renal physiology beyond basic memorization?	67	25	7	1	0	5 (4-5)
Did the numerical problem-solving complement your other methods for learning renal physiology?	63	27	8	1	1	5 (4-5)
Did you feel that your understanding of the volume of distribution, clearance, determination of GFR and RBF, and acid-base disturbances had improved, and that your confidence in applying these concepts had increased?	59	31	10	0	0	5 (4-5)
Was the numerical problem-solving exercise more engaging and interactive than traditional teaching methods?	70	17	12	1	0	5 (4-5)
Did you find the numerical problem-solving exercise sufficiently challenging?	43	32	20	5	0	4 (3-5)
Was the numerical problem-solving exercise clear and relevant to the topics covered in renal physiology?	60	32	8	0	0	5 (4-5)
Did the numerical problem-solving exercises encourage collaboration and discussion among peers to understand renal physiology concepts better?	63	23	13	1	0	5 (4-5)
Would you prefer to incorporate numerical problem-solving exercises more frequently throughout the renal physiology curriculum?	69	23	8	0	0	5 (4-5)
Do you believe numerical problem-solving exercises should be a standard part of future renal physiology curricula?	70	23	6	1	0	5 (4-5)

Additionally, 17 students responded to an open-ended question asking for “any other comments”. Most students appreciated the numerical exercise; a few demanded more numerical discussion classes. The two outstanding comments were “true experimental data and usage should be more like directly from the hospital. Detailed explanation of physiology along with quantitative calculation and prediction. Integration of all concepts and applying at a case study with open discussion”, and “I now have 17 questions to revise whenever I am worried about renal physiology. I am sure that will boost my confidence”.

## Discussion

The present study demonstrated that incorporating a numerical problem-solving module into renal physiology instruction improves post-test scores and is supported by students’ positive feedback, thereby validating the effectiveness of this pedagogical approach for teaching complex renal physiology concepts. We used an online audience response system (Google Forms) to administer pre- and post-test assessments. Previous studies have shown that students prefer MCQs delivered through an online audience response system [11,12].

The pre- and post-test MCQs were matched for difficulty and topic alignment. The significant improvement in both numerical and theoretical MCQ scores in our study suggests that the numerical module not only strengthened computational abilities but also enhanced broader conceptual understanding, indicating successful knowledge transfer between the mathematical and theoretical domains.

Item analysis of pre- and post-test MCQs provided valuable insights. A higher DifI indicates lower difficulty [10]. There was a small, non-significant increase in the mean DifI from pre-test to post-test, which may indicate that students’ comprehension improved on the post-test. The lower the DI, the greater the proportion of correct responses among low achievers, or the less the discrimination between low and high achievers [10]. The DI decreased non-significantly, which may indicate a narrowing of the knowledge gap between high and low achievers [10]. A non-significant reduction in DE indicates that students may have developed a more precise understanding, leading to fewer instances of conceptual confusion and, in turn, fewer instances of distractor use, rendering them non-functional.

The improvement in post-test scores was significant among low-achieving students, suggesting that numerical problem-solving exercises help bridge knowledge gaps and foster equitable learning outcomes. However, the lack of improvement in high-achieving students’ scores indicates the need to introduce more challenging exercises for this subgroup to further enhance their learning outcomes. A significant reduction in DE among pre-test low-achievers in the post-test suggests that these students selected distractors less often, indicating enhanced comprehension. This finding supports the principles of cognitive load theory [13]. The theory suggests that beginners benefit when intrinsic cognitive load is appropriately managed by activating prior knowledge, reducing unnecessary content, aligning material with their level of expertise, and progressing gradually from simple concepts to more complex topics [13].

The feedback from the Likert-scale questionnaire revealed several insights into the pedagogical value of the numerical problem-solving module. Consistently high median scores across most items indicate broad student acceptance and perceived usefulness of the intervention. Most students agreed or strongly agreed that the exercise reinforced their understanding of renal physiology, promoted deeper conceptual learning, and improved their confidence in applying physiological principles. These findings suggest that the module may have facilitated meaningful learning rather than superficial memorization. Notably, students strongly agreed that the module improved their understanding of complex concepts, including clearance, GFR, RBF, and acid-base disturbances. These topics are often considered difficult because they require integrating conceptual knowledge with quantitative reasoning. The positive responses, therefore, indicate that numerical exercises may help students bridge this gap more effectively.

Students also perceived the numerical problem-solving session as more engaging and interactive than didactic lectures. Strong agreement on peer collaboration further suggests that small-group numerical discussions fostered cooperative learning and academic interaction among students. Such interaction may promote deeper cognitive processing because students are required to explain their reasoning, defend their answers, and collectively interpret physiological data. This finding supports social constructivist theory [14], which holds that knowledge develops through social interactions and collaboration, as has also been proven by a recent study [15]. Students believed the module complemented their learning methods and facilitated comprehension, enabling them to apply the concepts more confidently.

Another important finding was that students considered the exercises sufficiently challenging while remaining relevant and understandable. This balance is educationally important because exercises that are too simple may fail to stimulate higher-order thinking. In contrast, those that are too difficult may reduce motivation and participation. The responses suggest that the module achieved an appropriate cognitive level for first-year MBBS students. Students believed the module should be a component of renal physiology curricula. Furthermore, the strong preference for incorporating numerical problem-solving more frequently into the renal curriculum reflects their recognition of the method’s value in bridging theoretical knowledge and practical application. The open-ended feedback emphasized the need to include more numerical questions based on real-world scenario data that integrate all concepts of renal physiology and to teach them through case studies.

To the best of the authors’ knowledge, studies with similar objectives are lacking for direct comparison; however, our results align with contemporary research in medical education. Roberts et al. (2016) found that first-year undergraduate medical students better understood complex renal physiology concepts through case-based discussions focused on clinical correlations with kidney physiology [2]. Another study used a redesigned multimodal renal physiology curriculum to help students move beyond rote memorization, promoting an understanding that students found enjoyable [16]. Xu et al. (2023) showed that integrating problem-based learning with lectures was more effective than lectures alone in teaching renal pathology to undergraduate students [6]. Another study from India found that changing traditional teaching methods for renal physiology is needed to build students’ confidence in applying their knowledge [17]. One review advocated using original experiments to teach renal physiology to cultivate stronger critical thinking in students [18]. Elzubeir (2012) showed that a problem-based renal curriculum allowed students to integrate basic anatomy and physiology with clinical renal problems. It was concluded that the problem-based renal curriculum effectively bridges the gap between basic sciences and clinical practice while maintaining high student motivation [19].

While previous studies have explored various innovative approaches to teaching renal physiology such as using podcasts [20], flipped classrooms [21,22], or classroom activities [23], our study uniquely focuses on the specific role of numerical problem-solving, filling a critical gap in the literature. The findings of this study demonstrate the value of integrating quantitative reasoning skills into medical education to prepare students for the increasingly data-driven nature of modern medical practice.

We suggest that numerical problem-solving be treated as a core component of renal physiology education rather than a supplementary tool. Although this module is not a wet-laboratory activity, it promotes analytical reasoning, physiological interpretation, quantitative clinical thinking, and applied problem-solving skills. Therefore, such sessions can reasonably be integrated into practical/tutorial hours, competency-based skill sessions, or formative assessment modules. This is particularly relevant in CBME curricula, where application, interpretation, and problem-solving skills are strongly emphasized.

The current study’s findings open several avenues for future research. First, longitudinal studies could examine the long-term effects of numerical problem-solving on clinical decision-making skills. Second, the integration of numerous real-world, scenario-based numerical problems, as suggested by student feedback, could be tested for effectiveness. Finally, the module’s scalability across other physiology topics or medical disciplines could be investigated. Future iterations of the module could integrate virtual simulations, dynamic physiological datasets, or clinical video scenarios to enhance experiential learning and further bridge the gap between theoretical physiology and clinical application.

Limitations

This study has several limitations. First, it was conducted at a single institution in a single session with a relatively small and convenient sample size, which may limit the generalizability of the findings to other medical schools. A multicenter validation with diverse student populations would strengthen the applicability of the results. Second, the study relied on anonymous feedback, preventing a direct correlation between students’ perceptions and performance outcomes. Third, the use of fewer numerical questions (only eight in the pre-test and six in the post-test) might have reliability issues for comparison of results. Fourth, the lack of long-term follow-up data means the study cannot assess whether the observed improvements in understanding and problem-solving skills are retained over time. Future research should include analyses of long-term knowledge retention, such as follow-up assessments conducted several months after the intervention.

Additionally, the Hawthorne effect, in which students may have performed better simply because they knew they were being observed, cannot be ruled out [24]. Finally, the study used a quasi-experimental design rather than a randomized controlled trial, limiting causal inference. Future studies employing controlled interventions with randomized participant groups would provide more definitive evidence on the effectiveness of numerical problem-solving in medical education. Methodological constraints require cautious interpretation of results.

## Conclusions

This study highlights the potential of numerical problem-solving to deepen understanding of renal physiology. While the results are promising, particularly for low-achieving students, refining the module to include more real-world scenarios and a more diverse group of participants could further validate its scalability and effectiveness. By addressing these areas, numerical problem-solving exercises could become a standard component of medical education, fostering analytical reasoning and clinical competence.

Nevertheless, the findings should be interpreted with caution. The study lacked a control group and employed only a short-term post-test assessment. Consequently, it remains unclear whether the observed improvements reflect sustained conceptual understanding or merely short-term recall.

## Appendices

Appendix A 

**Table 5:** Numerical problem-solving module in renal physiology d: day; dL: decilitre; g: gram; h: hour; HCO3-: bicarbonate; kg: kilogram; L: litre; mEq: milliequivalent; mg: milligram; min: minute; mL: millilitre; mmHg: millimetres of mercury; mOsm: milliosmoles; PaCO_2_: partial pressure of carbon dioxide; PaO_2_: partial pressure of oxygen; pH: potential of hydrogen

Numerical questions in renal physiology
1. Calculate the total body water for a 70 kg male from the given data: One hundred millicuries (mCi) of deuterium were injected. 4% of the injected deuterium was excreted after one hour. The deuterium plasma concentration at the end of one hour was 0.002 mCi/mL.
2. Calculate the extracellular fluid volume for a 70 kg male from the given data: 4 mg of inulin was injected. 1.2 mg of inulin was excreted in one hour. The plasma concentration of inulin at the end of one hour was 0.02 mg/dL.
3. A 70 kg man received an intravenous injection of 12 mg of Evans Blue dye. After equilibrium was reached, the dye concentration in his blood was 0.4 mg/dL. What is the volume of distribution of the dye?
4. Calculate the blood volume of a female who received an intravenous injection of 10 microcuries (μCi) of radio-iodinated serum albumin (RISA). A venous blood sample collected twelve minutes later showed a plasma RISA activity of 4 μCi/L. Her estimated packed cell volume was 40%.
5. Determine the direction of bulk flow and calculate the net pressure driving bulk flow using the following data: peritubular capillary hydrostatic pressure = 16 mmHg, tubule hydrostatic pressure = 6 mmHg, peritubular capillary osmotic pressure = 28 mmHg, tubule osmotic pressure = 0 mmHg.
6. In an animal experiment focusing on a single nephron of the kidney, the following were observed: The glomerular filtration rate was 42 nanolitres per minute. The hydrostatic pressure in the glomerular capillary and Bowman’s/Malpighian capsule was 50 mmHg and 12 mmHg, respectively. The oncotic pressure in the glomerular capillary and Bowman’s/Malpighian capsule was 24 mmHg and 0 mmHg, respectively. Calculate the glomerular ultrafiltration coefficient.
7. Calculate the inulin clearance, given that the concentrations of inulin in urine and plasma were 29 mg/mL and 0.25 mg/mL, respectively, and a 60 mL/h urine flow rate.
8. Calculate the tubular load of substance ‘X’ with a given plasma concentration of 0.4 mg/dL and a glomerular filtration rate of 150 mL/min, assuming ‘X’ is freely filtered and experiences neither secretion nor reabsorption in the tubules.
9. Determine the excretion rate of substance ‘X’, considering its concentration in urine was 0.1 mg/mL and the urine flow rate was 2 mL/min.
10. Calculate the percentage of sodium excreted by a 40-year-old healthy man who was administered a thiazide diuretic, and the following data were recorded from the clearance study: Inulin concentration in plasma: 1 mg/mL; Inulin concentration in urine: 10 mg/mL; Sodium concentration in plasma: 140 mEq/L; Sodium concentration in urine: 140 mEq/L; Flow rate of urine: 10 mL/min.
11. Determine whether the freely filterable substance ‘X’ undergoes reabsorption or secretion, considering the following data: Plasma concentration of X was 0.5 mg/mL; Glomerular filtration rate was 110 mL/min; The concentration of X in urine was 15 mg/mL; The flow rate of urine was 4 mL/min. Additionally, calculate the rate of reabsorption or secretion.
12. Calculate the actual renal blood flow for a 35-year-old female suspected of renal disease using the following data obtained during the clearance study: Arterial concentration of para-aminohippurate (PAH): 6.0 mg/dL; Renal vein concentration of PAH: 1.0 mg/dL; Urine concentration of PAH: 5.0 mg/mL; The flow rate of urine: 3.0 mL/min; Packed cell volume: 50%.
13. In an experiment, a 60-kg male received an injection of one gram of mannitol. Upon reaching equilibrium, 20% of the injected mannitol was excreted, and the plasma concentration of mannitol stabilised at 0.08 g/L. Which fluid compartment volume can be estimated using mannitol? Calculate this volume based on the provided data.
14. For a person suffering from severe diabetes insipidus, the hypothetical urine osmolarity in milliosmoles (mOsm) per litre of urine formed and urine flow rate per day could be: a. 150 mOsm/L, 12 L/d; b. 900 mOsm, 2 L/d; c. 150 mOsm/L, 2 L/d; d. 900 mOsm/L, 12 L/d
15. Calculate the minimum volume of urine that should be formed for a 70-kg male with chronic kidney disease if he had to excrete 600 mOsm of solute per day in urine. The maximum urine concentrating ability of his kidneys is 900 mOsm/kg of water.
16. Which set of plasma osmolality, urine osmolality, and plasma sodium concentration (listed in that order in the options) should raise suspicion of the syndrome of inappropriate antidiuretic hormone secretion? a. 300 mOsm/kg, 600 mOsm/kg, 140 mEq/L; b. 270 mOsm/kg, 900 mOsm/kg, 130 mEq/L; c. 300 mOsm/kg, 300 mOsm/kg, 140 mEq/L; d. 320 mOsm/kg, 200 mOsm/kg, 130 mEq/L
17. A 55-year-old man with diabetes mellitus presents to the emergency department with 24 hours of weakness, confusion, and nausea. He appears lethargic and is breathing rapidly on examination. Arterial blood gas analysis result shows the following: pH: 7.28; PaCO2: 30 mmHg; PaO2: 96 mmHg; HCO3-: 15 mEq/L. Based on these findings, what is the most likely acid-base balance disorder in the patient?
18. A 30-year-old female presents to the emergency department with tingling in her hands and feet, as well as occasional muscle cramps. She reports increased anxiety and hyperventilation over the past three days. Arterial blood gas analysis test reveals the following: pH: 7.50; PaCO2: 25 mmHg; PaO2: 95 mmHg; HCO3-: 18 mEq/L. Based on these findings, what is the most likely acid-base balance disorder in the patient?
19. A 65-year-old man with a history of chronic obstructive pulmonary disease arrives at the emergency department with increasing shortness of breath and confusion over the last two days. He has not been adhering to his medications or oxygen therapy. On examination, he appears somnolent, with a respiratory rate of 10 breaths per minute. Arterial blood gas analysis result shows the following: pH: 7.25; PaCO2: 55 mmHg; PaO2: 85 mmHg; HCO3-: 38 mEq/L. Based on these findings, what is the most likely acid-base balance disorder in the patient?
20. A 55-year-old female presents to the clinic with muscle cramps, weakness, and a history of frequent vomiting over the past two weeks due to a gastrointestinal illness. On examination, her respiratory rate is 8 breaths per minute. Arterial blood gas analysis test reveals the following: pH: 7.50; PaCO2: 50 mmHg; PaO2: 88 mmHg; HCO3-: 44 mEq/L. Based on these findings, what is the most likely acid-base balance disorder in the patient?

Appendix B 

**Table 6:** Multiple-choice questions used in pre- and post-test dL: decilitre; g: gram; HCO3-: bicarbonate; kg: kilogram; L: litre; mEq: milliequivalent; mg: milligram; min: minute; mL: millilitre; mmHg: millimetres of mercury; mOsm: milliosmoles; PaCO_2_: partial pressure of carbon dioxide; PaO_2_: partial pressure of oxygen; pH: potential of hydrogen

Pre-test multiple-choice questions	Post-test multiple-choice questions
The kidneys are crucial for maintaining acid-base balance by removing hydrogen ions. Which type of renal tubule cell primarily performs this function? a) Principal cells; b) Alpha intercalated cells; c) Beta intercalated cells; d) Mesangial cells	How do the proximal convoluted tubule (PCT) and the collecting duct (CD) differ in structure and function? a) The PCT has a brush border, unlike the CD; b) Type II carbonic anhydrase is present in the PCT but not in the CD; c) The PCT has tight junctions, whereas the CD’s junctions are more permeable; d) Potassium-sparing diuretics target the PCT, whereas osmotic diuretics primarily affect the CD
Juxtaglomerular nephrons are a specialised type of nephron with distinct characteristics. Which statement about these nephrons is correct? a) Oxygen consumption is higher than in cortical nephrons; b) Blood flow is greater than in cortical nephrons; c) They can produce more concentrated urine than cortical nephrons; d) The peritubular capillary network is shorter than in cortical nephrons	Which structure is typically NOT found in the renal medulla? a) Juxtaglomerular apparatus; b) Loop of Henle; c) Collecting duct; d) Vasa Recta
Which of the following would most likely increase after dilation of the efferent arterioles in the kidney? a) Renal blood flow; b) Glomerular capillary hydrostatic pressure; c) Filtration fraction; d) Hydrostatic pressure in Bowman’s space	Which statement best describes renal autoregulation? a) It involves increased renal vascular resistance as mean arterial pressure decreases from 100 to 80 mmHg; b) It primarily involves changes in the diameter of the efferent arteriole; c) It effectively preserves normal renal blood flow during hypotension when arterial pressure drops below 50 mmHg; d) It reduces the impact of arterial blood pressure fluctuations on renal sodium excretion
When the glomerular filtration rate increases, the proximal tubule exhibits a corresponding increase in sodium reabsorption. This phenomenon is known as: a) Autoregulation; b) Tubuloglomerular feedback; c) Saturation of tubular transport; d) Glomerulotubular balance	A 30-year-old male undergoes routine renal function testing. His results are as follows: glomerular hydrostatic pressure: 50 mmHg; Bowman’s capsule hydrostatic pressure: 12 mmHg; glomerular oncotic pressure: 24 mmHg; ultrafiltration coefficient: 10 mL/min/mmHg. Based on these data, what is his estimated glomerular filtration rate in mL/min? a) 100; b) 120; c) 140; d) 160
A 35-year-old male’s renal haemodynamic assessment reveals: hydrostatic pressure in the glomerular capillary is 55 mmHg, hydrostatic pressure in Bowman’s space is 15 mmHg, oncotic pressure in the glomerular capillary is 30 mmHg, and oncotic pressure in Bowman’s space is 0 mmHg. What is the patient’s net filtration pressure in mmHg? a) 5; b) 10; c) 15; d) 20	A 45-year-old male undergoes a comprehensive renal function assessment. Given the following results: Para-amino hippurate clearance = 600 mL/min; Inulin clearance = 120 mL/min. What is his approximate filtration fraction? a) 0.10; b) 0.20; c) 0.30; d) 0.40
A 68-year-old male with chronic kidney disease has the following laboratory results: plasma creatinine = 2.0 mg/dL; urine creatinine = 100 mg/dL; urine flow rate = 1.5 mL/min. What is his estimated creatinine clearance (mL/min)? a) 25; b) 50; c) 75; d) 100	A 35-year-old man with nephrotic syndrome has a serum albumin level of 2.0 g/dL (normal: 3.5-5.5 g/dL) and noticeable swelling in his limbs. Which of the following best describes the underlying cause of his oedema? a) Increase in capillary hydrostatic pressure; b) Increase in interstitial hydrostatic pressure; c) Decrease in plasma oncotic pressure; d) Increase in lymphatic drainage
Sodium reabsorption is vital for maintaining fluid and electrolyte balance in the kidneys. Which nephron segment primarily uses luminal membrane sodium channels for sodium reabsorption? a) Proximal tubule cells; b) Thick ascending limb cells; c) Distal convoluted tubule cells; d) Collecting duct principal cells	A 63-year-old male with heart failure begins furosemide therapy. After one week, he reports weakness and muscle cramps. Which electrolyte change is most likely to be observed in this patient’s plasma after starting a loop diuretic? a) Increased sodium; b) Decreased potassium; c) Increased chloride; d) Decreased bicarbonate
Substance ‘X’ is freely filtered by the kidneys. Given a plasma ‘X’ concentration of 2 mg/mL, a glomerular filtration rate of 100 mL/min, a urine ‘X’ concentration of 10 mg/mL, and a urine flow rate of 5 mL/min, we can conclude that the kidney tubules: a) reabsorb ‘X’ at 150 mg/min; b) reabsorb ‘X’ at 200 mg/min; c) secrete ‘X’ at 150 mg/min; d) secrete ‘X’ at 200 mg/min	A 55-year-old woman undergoes renal function testing. Her inulin clearance is 95 mL/min, and her creatinine clearance is 105 mL/min. What explains the discrepancy between these values? a) Partial reabsorption of inulin in the renal tubules; b) Creatinine is secreted in the renal tubules; c) Inulin is metabolised in the renal tubules; d) Creatinine is synthesised in the renal tubules
A 50-year-old diabetic male’s laboratory results show: plasma glucose concentration, 400 mg/dL; glomerular filtration rate, 100 mL/min; and maximum tubular glucose reabsorption, 375 mg/min. What is his urinary glucose excretion rate in mg/min? a) 0; b) 25; c) 50; d) 100	A 35-year-old diabetic male with a fasting plasma glucose of 320 mg/dL has a renal glucose clearance of 10 mL/min. Which explanation best accounts for the presence of glucose in his urine? a) Renal glucose reabsorption threshold exceeded; b) Defective proximal tubular glucose reabsorption; c) Enhanced renal glucose reabsorption; d) Reduced filtered glucose load due to low glomerular filtration rate
A patient with congestive heart failure experiences significant sodium retention by the kidneys. What is the likely cause? a) Decrease in effective circulating blood volume; b) Decrease in extracellular fluid volume; c) Decrease in total blood volume; d) Increase in total blood volume	A 60-year-old male with hypotension and tachycardia receives 2 litres of intravenous saline after a motor vehicle accident. What is the expected distribution of the fluid in his body? a) Equal expansion of intracellular fluid and plasma volume; b) Expansion of extracellular fluid with minimal effect on intracellular volume; c) Predominant expansion of plasma volume with minimal effect on interstitial fluid; d) Uniform expansion across intracellular, interstitial, and plasma compartments
A 25-year-old male drinks 1.5 litres of water in the morning. Which renal changes are most likely to occur after this significant fluid intake? a) Increased renin and aldosterone secretion; b) Reduced antidiuretic hormone release and higher urine osmolality; c) Increased atrial natriuretic peptide release with increased sodium excretion; d) Decreased glomerular filtration rate and increased water reabsorption	A healthy 30-year-old male receives 2 litres of intravenous isotonic saline during a medical procedure. Which renal changes are most likely to occur in this patient after the intravenous saline infusion? a) Decreased renin and decreased atrial natriuretic peptide; b) Decreased renin and increased glomerular filtration rate; c) Increased antidiuretic hormone release and increased urine osmolality; d) Increased aldosterone and increased sodium reabsorption
An elderly male has the following renal parameters: effective renal plasma flow by PAH clearance: 600 mL/min; haematocrit: 40%. What is the estimated renal blood flow in mL/min? a) 600; b) 900; c) 1000; d) 1500	A 65-year-old hypertensive male undergoes a para-aminohippurate (PAH) clearance study to estimate renal plasma flow (RPF). The following measurements were obtained: arterial concentration of PAH, 5 mg/dL; PAH concentration in renal vein, 0.5 mg/dL; urine PAH concentration, 15 mg/mL; urine flow rate, 1.8 mL/min. What was his RPF in mL/min? a) 360; b) 540; c) 600; d) 750
A 70-year-old man with syndrome of inappropriate antidiuretic hormone secretion presents with the following lab findings: urine osmolality of 600 mOsm/L, plasma osmolality of 300 mOsm/L, and urine flow rate of 2 mL/min. What is his free water clearance in mL/min? a) -2.0; b) -1.0; c) 0; d) 2.0	A 47-year-old woman presents with polyuria and polydipsia. Suspecting diabetes insipidus, her doctor orders a urine concentration test. The results show a urine osmolality of 100 mOsm/kg, a plasma osmolality of 300 mOsm/kg, and a urine flow rate of 15 mL/min. What was her free water clearance in mL/min? a) -2; b) 0; c) 5; d) 10
A 68-year-old man with severe chronic obstructive pulmonary disease develops increasing lethargy and confusion. His arterial blood gas analysis results are: pH: 7.28, PaCO₂: 60 mmHg, and HCO3-: 30 mEq/L. Which best characterizes his acid-base status? a) Acute respiratory acidosis; b) Chronic respiratory acidosis with metabolic compensation; c) Metabolic acidosis with respiratory compensation; d) Mixed metabolic and respiratory acidosis	A 55-year-old woman presents with a three-day history of severe vomiting and anxiety-induced hyperventilation. Blood gas analysis shows a pH of 7.5, PaCO2 of 32 mmHg, and HCO3- of 36 mEq/L. Which option best explains the acid-base disturbance in this case? a) Mixed metabolic and respiratory alkalosis; b) Acute metabolic alkalosis with respiratory compensation; c) Acute respiratory acidosis with metabolic compensation; d) Respiratory alkalosis with metabolic compensation
A 55-year-old woman presents to the emergency department with a three-day history of severe vomiting. Arterial blood gas results show a pH of 7.50, PaCO2 of 47 mmHg, and HCO3- of 38 mEq/L. Which option best explains her acid-base disturbance? a) Acute metabolic alkalosis with respiratory compensation; b) Acute respiratory acidosis with metabolic compensation; c) Mixed metabolic alkalosis and respiratory acidosis; d) Respiratory alkalosis with metabolic compensation	A 35-year-old climber at 14,000 feet develops acute mountain sickness. Blood gas analysis shows: pH 7.48, PaCO2 30 mmHg, and HCO3- 20 mEq/L. What is the primary renal response to these high-altitude physiological changes? a) Increased bicarbonate reabsorption; b) Increased ammonia excretion; c) Decreased bicarbonate reabsorption; d) Increased hydrogen ion secretion
The kidneys can remarkably concentrate urine, conserving water. This process relies on a complex mechanism called the countercurrent multiplier. Which of the following structures is not a key component of the countercurrent multiplier system? a) Thick ascending limb of the loop of Henle; b) Collecting duct; c) Vasa recta; d) Thin descending limb of the loop of Henle	The kidneys maintain a high concentration of solutes in the renal medulla, which is essential for water reabsorption and the production of concentrated urine. Which mechanism is crucial for maintaining this renal medullary osmotic gradient? a) High permeability of the ascending limb of the loop of Henle to water; b) Slow blood flow through the vasa recta to prevent solute washout; c) Impermeability of the descending limb of the loop of Henle to sodium ions; d) Impermeability of the inner medullary collecting duct to urea
The vasa recta help preserve the medullary osmotic gradient by: a) Actively transporting sodium ions into the interstitial fluid; b) Facilitating countercurrent exchange to reduce solute washout; c) Secreting antidiuretic hormone to enhance water reabsorption; d) Filtering plasma proteins to maintain oncotic pressure in the medulla	Sodium reabsorption is a crucial process in the kidney, essential for maintaining fluid and electrolyte balance. Which of the following nephron segments is the primary site for sodium reabsorption? a) Proximal convoluted tubule; b) Distal convoluted tubule; c) Thick descending Loop of Henle; d) Collecting duct

Appendix C

1. Did solving numerical problems reinforce your overall understanding of renal physiology?

2. Did the numerical problem-solving exercise enable deeper exploration of renal physiology beyond basic memorization?

3. Did the numerical problem-solving complement your other methods for learning renal physiology?

4. Did you feel that your understanding of the volume of distribution, clearance, determination of GFR and RBF, and acid-base disturbances had improved, and that your confidence in applying these concepts had increased?

5. Was the numerical problem-solving exercise more engaging and interactive than traditional teaching methods?

6. Did you find the numerical problem-solving exercise sufficiently challenging?

7. Was the numerical problem-solving exercise clear and relevant to the topics covered in renal physiology?

8. Did the numerical problem-solving exercises encourage collaboration and discussion among peers to understand renal physiology concepts better?

9. Would you prefer to incorporate numerical problem-solving exercises more frequently throughout the renal physiology curriculum?

10. Do you believe numerical problem-solving exercises should be a standard part of future renal physiology curricula?

Open-ended question: Any other comments?
